# A balancing act–finding one´s way to health and well-being: A qualitative analysis of interviews with Swedish university students on lifestyle and behavior change

**DOI:** 10.1371/journal.pone.0275848

**Published:** 2022-10-13

**Authors:** Katarina Åsberg, Ann Catrine Eldh, Marie Löf, Marcus Bendtsen

**Affiliations:** 1 Department of Health, Medicine and Caring Sciences, Linköping University, Linköping, Sweden; 2 Department of Public Health and Caring Sciences, Uppsala University, Uppsala, Sweden; 3 Department of Biosciences and Nutrition, Karolinska Institute, Solna, Sweden; University College London, UNITED KINGDOM

## Abstract

**Introduction:**

Unhealthy lifestyle behaviors such as unhealthy diets, low physical activity levels, smoking, and harmful alcohol consumption are common in student populations, which constitute a large group of young adults. As unhealthy lifestyle behaviors are associated with future disease and premature mortality, most commonly from cardiovascular disease and cancers, it is from a public health perspective important to understand such behaviors in young adult populations. The objective of this study was to investigate university students’ experiences of health, health-related behaviors, and the barriers and facilitators for behavior change in terms of health promotion in everyday life.

**Materials and methods:**

This qualitative study was conducted at a middle-sized university in Sweden. Students represented different faculties and were recruited via non-probability convenience sampling using means such as the snowball technique and social media. The 21 interviews with 24 students, individually or in groups, were transcribed verbatim prior to a qualitative analysis inspired by phenomenological hermeneutics.

**Results:**

Our interviews showed that university student life is associated with new health-related challenges, for example study-related stress and procrastination implies a lack of energy to engage in healthy routines such as physical activity, and a limited budget affects food choices. While adapting to a new context, students explore personal strategies such as taking on changes in manageable steps, seeking social support, and avoiding disturbances to maintaining health and quality of life.

**Conclusions:**

Experiences of health while becoming and being a university student can be described as a transition–a balancing act of walking a slack line–during which students seek to manage a healthy balance. In the past, interventions have to some extent been designed to address university students’ behaviors; however, our study aids an understanding of their needs. Future interventions should highlight the transitions they are experiencing and the challenges of student life.

## Introduction

University students constitute the single largest sub-group of young adults (18–25 years) in Sweden. In 2021, more than one third of all young adults (approximately 380 000) were registered at the 35 universities across the country. Becoming a university student often involves changes to one’s way of living and, for some, this includes developing or sustaining unhealthy lifestyle behaviors [1–3].

Unhealthy lifestyle behaviors such as unhealthy diets, low physical activity levels, smoking, and harmful alcohol consumption are known to increase the risk of noncommunicable diseases (NCDs), such as cancer and cardiovascular disease. If the prevalence of these behaviors were to decrease, the burden of disease caused by NCDs could be greatly reduced [4]. As unhealthy lifestyle behaviors are common in student populations worldwide [5–8], and are associated with future disease and premature mortality [9–13], it is from a public health perspective important to further understand these behaviors in young adult populations.

Although there is insufficient public health data regarding Swedish students’ lifestyle behaviors, the highest alcohol consumption and smoking behaviors in the population are noted among 18–29-year-olds [14]. Furthermore, many international studies have investigated lifestyle and self-perceived health among university students, mainly using cross-sectional studies. For example, Pengpid et al. [15] surveyed more than 12 000 university students from 24 countries regarding their sedentary behavior, physical activity, life satisfaction and happiness. They found that higher sedentary behavior (4 to <8h and ≥8 hours) was associated with poorer life satisfaction and lower happiness, while moderate and/or high physical activity increased the odds for greater happiness and higher life satisfaction. In addition, a review by Antonopoulou et al. [16] showed that students’ self-reported adherence to a Mediterranean diet adherence was correlated with a lower risk for depression, while Arsandaux et al.’s review on self-esteem and health risk behaviors such as: substance use, sexual behavior, physical activity, and nutrition among students, identified higher self-esteem to be associated with healthier behavior [17].

Besides studies on health and lifestyle behavior, there are plenty of intervention studies conducted in student and young adult populations, ranging from face-to-face interventions to digital interventions, targeting for example: alcohol consumption, smoking, physical activity and dietary behaviors [18, 19], sleep [20], and/or sexual [21] and mental health [22]. Several interventions have shown promising results in facilitating improvements of unhealthy lifestyle behaviors among university students.

Despite the many studies been conducted, there are gaps in the evidence base. Plotnikoff et al. [19] examining the effectiveness of interventions targeting physical activity, nutrition, and weight-loss behaviors among university students, highlight that participant numbers were notably small and most participants female in the included studies. Also, the number of qualitative studies investigating university students´ lived experience and preconceptions on multiple lifestyle and behavior change is limited [23–26].

An expanded understanding of university students’ health perspectives can enable a further development of appropriate interventions and implementation strategies guidance for this large group of young people to find a path to health and well-being. The objective of this study was to investigate university students’ experiences of health, health-related behaviors, and barriers and facilitators for behavior change in terms of health promotion in everyday life.

## Materials and methods

Interviews, both individual and one group interview, were conducted with 24 university students, and analyzed using qualitative analysis inspired by phenomenological hermeneutics [27]. The manuscript comprises applicable items from The Standards for Reporting Qualitative Research (SRQR) [28]. The phenomenological hermeneutical method for interpreting interview text, by Lindseth and Norberg [27, 29] is inspired by the theory of interpretation presented by philosopher P. Ricoeur. It is a method for elucidating the meaning of life world phenomena by researching lived experience, in this case university students’ understanding of health and health behaviors in everyday life.

### Study design and recruitment

This qualitative study was carried out at a middle-sized (32 400 students) university in Sweden. The university offers a broad range of education, including technical, philosophical, and medical programs and courses at all levels from undergraduate, to post graduate and PhD levels. Like all Swedish universities, there are no educational fees, and students can apply from across Sweden. Students at undergraduate level from the technical, philosophical, and medical faculties were invited to either individual or group interviews according to their preference. Inclusion criteria were: being a university student, aged 18 years or older, and speaking Swedish.

Students were recruited via non-probability convenience sampling and the study was advertised through several means: (1) posters in communal areas on the two largest campuses; (2) digital broadcasts via the university and student union website and social media; (3) face-to-face recruitment on campus and (4) snowball sampling to approach contacts of interviewees. Students registered their interest by contacting the first author by email. An interview date was agreed upon via email and written study information was emailed to each student. At the same time, students were also sent information about the services available to them should they wish to seek health-related support, including contact details for a project-independent contact person at the student health center.

Before each interview, the interviewer ensured that the student had had the opportunity to read and understand the background information, including voluntariness and the right to withdraw at any time without any need to justify such a decision. Thereafter, the student was asked if they consented to take part, and verbal informed consent was recorded for each person. Subsequently, and prior to the main interview questions, they were asked to respond to three demographic questions: sex, age, and educational program.

All procedures involving participants were conducted in accordance with the ethical standards of the national research committee and with the 1964 Helsinki Declaration and its later amendments. This study was approved by the Swedish Ethical Review Authority on September 7, 2020 (Dnr 2020–02937). The one risk identified was the potential that interviewees might experience anxiety or health-related concerns due to participating in an interview. Consequently, the information disseminated to all participants prior to the interview included which resources that were available at the university for health-related support.

All 24 students who made contact chose to take part. Altogether, 21 interviews were conducted: 20 individual interviews and one group interview with four participants. Recruitment of students continued until data saturation was reached in terms of code and meaning saturation [30]. At 19 interviews, saturation was assumed, and this was confirmed in the two additional, and final, interviews. Participant demographics are shown in Table 1. The mean age of the students was 24 years (range: 19–44 years) and 67% (n = 16) were women. A majority were studying in the medical faculty (n = 17, 71%), followed by the technical (n = 4, 17%), and philosophical (n = 3, 12%) faculties.

**Table 1:** Participant characteristics.

Characteristics	Participants (n = 24)
**Sex**	
Women	16 (67%)
Men	8 (33%)
**Mean age (range)**	24 (19–44)
**Faculty**	
Medical	17 (71%)
Technical	4 (17%)
Philosophical	3 (12%)

### Data collection

All interviews were conducted during October to December 2020, via a digital conference system, and audio recorded. A study-specific interview guide, available in S1 File, was used for both the individual and group interviews. This consisted of open-ended questions which enabled the students to describe and reflect on their lifestyle, health-related behavior, and lifestyle behavior change.

All interviews were conducted by the first author who has a background in public health. Besides previous experience in conducting qualitative interviews, there was also an expert in the research team, providing guidance. The interviewer had no previous or additional contact or relationship with the students. The interviews lasted for a mean time of 46 minutes (range: 33–72 minutes). The interviews were transcribed verbatim by a skilled secretarial service specially procured, resulting in 405 pages of single-spaced text.

### Data analysis

Phenomenological hermeneutics [27] inspired the analysis of the university students’ understanding of the phenomena of health and health behaviors in everyday life. Data analysis of the verbatim transcripts followed the three methodological steps suggested by Lindseth and Norberg [29]: (1) naïve reading, (2) structural analysis, and (3) comprehensive understanding. In more detail, these steps involved the following:

1. Each interview transcript was read several times to grasp its overall meaning, composed as a naïve understanding. When all the interviews had been subjected to this procedure, all naïve understanding texts were read separately and jointly, to form a common naïve understanding of the entire dataset.
2. A subsequent structural analysis was performed, employing an inductive approach, as suggested by Elo and Kyngäs [31]. The first phase comprised open coding for meaning units corresponding to the study’s aim, using NVivo 12 Plus, across all interview transcripts. This identified 174 codes. By considering definitions of the codes and the meaning units represented, 42 subcategories were formed. During the further analysis, these 42 subcategories were abstracted into seven categories. The naïve understanding summary was used as a backdrop during this phase, to ensure consistency throughout the analysis and adherence to the study’s aim.
3. To conclude, a comprehensive understanding was summarized and reflected in relation to the study’s aim and the overall context of being a university student.

The analysis was performed by the first author (KÅ), with the second (ACE) and last (MB) co-authors reading and discussing all steps of the analyses, including how codes were extracted and defined, the forming of subcategories and categories, and thus the emerging findings. The comprehensive understanding was discussed in light of the naïve understanding and the structured analysis. In the event of queries regarding consistency and authenticity, a collaborative effort was made until agreement was reached on how best to understand the data [29]. In summary, an interdisciplinary group of authors with various research and professional competences in public health and qualitative methods participated in the analysis.

## Results

To ensure transparency, all three steps of the findings are reported in the result section, starting with the 1) naïve understanding, followed by the outcomes of the 2) structured analysis, and concluding with the 3) comprehensive understanding.

### Naïve understanding

The university years constitute a period that offers many opportunities for personal development and learning how to take care of oneself and a household. Initially, social interactions, friendships, and becoming part of the student community are the top priorities, but gradually one becomes more independent. The community influences one’s attitudes, perceptions, and choices relating to health and lifestyle. One wants to feel good and succeed with one’s studies, while still seizing the opportunity to have fun. However, one is also aware of influences that affect one’s health and well-being, even if one does not talk about them in everyday life. Everyone knows what is good for them, but then one wants something quick and easy that fits into everyday life, and in the end one’s finances determine the choices that are made. Studying takes a lot of time, and it is difficult to prioritize good habits during stressful periods. One wants to feel good yet have the energy to study and perform well academically. Behavior change requires one to be motivated; it has to be of importance to oneself, and one must be willing to change.

### Structural analysis

The seven categories relating to the lived experience of university students, formed by the 42 subcategories, are presented in Table 2 and further illustrated below.

**Table 2:** Sub- and main-categories from students’ views and experiences of health-related behavior and behavior change.

**Subcategories**	**Main categories**
Life-changing experience of becoming a student	Becoming a student
Wanting to become part of the student community	
Drinking alcohol is part of student culture	
Realizing that personal maturity comes with age and study years	
Feeling pleased with coherency being meaningful and stimulating	
Managing a limited budget	New student-related challenges
Having freedom with responsibility	
Having difficulties prioritizing health when studies take precedence	
Struggling to find study technique and discipline	
Finding the mobile phone both vital and stressful	
Noticing changes in eating habits	
Having experience of how sleeping hours affect studies and well-being	
Dealing with studies being very sedentary	
Feeling guilty if not meeting society’s health norms	Exposed to and influenced by others
Sensing health-related pressure from friends and family	
Being affected by the social network	
Feeling both negatively affected and inspired by social media	
Dealing with health information knowledge	
Having experienced healthy habits in childhood shapes behavior	
Wanting to feel good but it is not something thought of daily	Seeking a balance in health behaviors
Feeling a need for freedom to choose behaviors	
Believing that what one does regularly is the meaning of lifestyle behaviors	
Expressing that one’s lifestyle can say something about who one is	
Having experienced that lifestyle behaviors affect each other	Comprehending behavior change
Having experienced that behavior change fluctuates back and forth	
Having experienced that behavior change is difficult	
Seeing an urge for quick fixes when making changes	
Perceiving consequences affects motivation and behavior	
Having experienced that performance thinking can either inhibit or trigger	
Knowing that it is a prerequisite that oneself wants change	Implying behavior change
Seeing that driving forces and motivation are individual	
Wanting to perform and succeed with studies	
Wanting something of value, or avoiding something negative	
Wanting to improve, develop, and get better	
Wanting to be trustworthy as a future health professional	
Succeeding in change creates pride and feelings of success	
Implementing the change in manageable steps	Managing change in everyday life
Participating in challenges and competition with others	
Seeking social support, live or on the internet	
Making food plans, preparations, and deciding in advance to say no	
Making plans and schedules for implementing physical activity	
Removing notifications and apps as a way of decreasing mobile phone use	

#### Becoming a student

The interviewees described becoming a student as a life-changing experience, dealing with new responsibilities, leaving their previous circle of friends and family, and experiencing difficulties keeping up with their lifestyle behaviors while establishing new routines.

They shared experiences of initially wanting to become part of the student community: succeeding in building social relationships was nearly as important as the studies themselves. The many opportunities to take part in social activities, mostly including large amounts of alcohol, were described as a natural way to build social networks. However, later in their studies, the students noticed a gradual reduction in their own alcohol consumption and a decreased interest in regularly attending social events.

#### New student-related challenges

The students were exposed to study-related stress, and the mental strain entailed difficulties in prioritizing healthy routines. A lack of time and control over the workload was mentally exhausting, leaving no energy to engage in health-related behaviors, including physical activity and preparing home-cooked meals. A limited student budget affected daily decisions, such as whether to buy vegetables, to choose cheaper alternatives, or whether to order take-away.

Students also described having struggled with procrastination, leaving them with a sense of failure and stress about unfulfilled commitments. They remarked upon being responsible for their own health and well-being, and that one had to shape one’s own routines to feel good, for instance getting up in the morning, going to bed at regular times, and being able to manage distractions from one’s mobile phone.

#### Exposed to and influenced by others

The students described having learnt earlier in life about the significance of a healthy lifestyle and the consequences of being unhealthy. They considered this knowledge universal and the way they thought about health-related behavior was linked to a sense of guilt about not ‘acting healthily’. Consequently, being exposed to health messages was described as potentially stressful, regardless of whether one followed the advice or not.

Students described having observed an ongoing healthy-lifestyle trend in society, in their social networks, and on social media. Fellow students’ commitments to being healthy had influenced their own behavior, and social media had brought both negative and positive emotions, including guilt and disappointment, but also inspiration. At times, students had used social media to access tips, know-how, and motivation for behavior change.

#### Seeking a balance in health behaviors

‘Lifestyle behaviors’ were described by the interviewees as activities or routines in which one engages on a regular basis without paying too much attention to them, including the number of hours one usually sleeps at night. Furthermore, ‘lifestyle’ was considered to be a value-based way of living, i.e., one’s lifestyle expresses one’s identity, for example being vegan, an athlete, or a ‘party animal’. The concept of health was described as largely absent from conversations with others. Instead, the students described talking about health-related topics, such as dinner plans, staying in shape, or going to the gym.

Students had sought a balance between wanting to care for themselves, to feel good, and be healthy, yet also wanting freedom, pleasure, and fun. They had made their health-related decisions by weighing up options from both health and pleasure perspectives.

#### Comprehending behavior change

The students explained that attempts to change one’s behavior were difficult, in particular when trying to integrate changes into everyday life. Some behaviors were considered more challenging to change than others, for instance to quit smoking in comparison to eating more healthily. Changes in different lifestyle behaviors were also described as linked; for instance, when exercising, one also became more motivated to eat healthily.

Lifestyle behaviors were considered to have been inconsistent over time, alternating between periods of healthy and less healthy behaviors. Deciding to become healthier had entailed a highly conscious shift of focus, while reverting back to unhealthy behaviors had been a more unconscious process, with a gradual fading back to an unhealthier way of living.

Students described their motivation for behavior change as related to the perceived negative consequences of an unhealthy lifestyle. Some had experienced negative physical and mental consequences, including bodily changes, mood swings, lack of energy, problems sleeping, and difficulties concentrating, which had made them consider behavior changes. On the other hand, those who had not experienced any negative consequences had not given serious thought to changing their behavior.

#### Implying behavior change

It was suggested that the driving forces and motivation behind behavior change were highly individual, and various students took different routes to reach the same goal. Commonly, one had to see a need for change, and want to change oneself, for any behavior change to take hold. Students described how having successfully changed their behavior had induced feelings of pride, pushed their boundaries, revised their self-images, and built self-esteem.

The students who had engaged in healthy lifestyle behaviors were motivated by a desire to perform well in their studies, to have more energy, to look and feel physically and mentally well, or to be emotionally rewarded by cooking or exercising. Long-term goals, such as wanting to be trustworthy as a future health professional, or a desire to avoid illness later in life, were other motivational aspects.

#### Managing change in everyday life

The students explained that managing health behavior change had included strategies of implementing change in manageable steps, seeking social support, planning, and avoiding disturbances.

Manageable steps were described in terms of having replaced certain meals with healthier alternatives, or gradually becoming more physically active. Seeking social support from others was perceived as a powerful motivator, either by taking on challenges or activities together, or sharing thoughts and encouraging each other.

The students also remarked that part of avoiding disturbances was a desire to decrease mobile phone screen time, since it had taken valuable time away from other activities. To decrease phone-related stress, the students had employed various strategies, including removing notifications and apps, hiding their phone out of sight, monitoring screen time, using silent mode, or deciding not to answer text messages immediately.

### Comprehensive understanding

Becoming and being a university student is a balancing act. Adapting to a new context, while simultaneously seeking strategies for good health and well-being, means that a desire to make informed health choices that contribute to academic performance is balanced against enjoying student life.

As a student, one faces new health-related challenges over the course of one’s academic studies, and students respond to these challenges by exploring and developing personal strategies to maintain their health and quality of life. Lifestyle behavior change is an emotional process that can be both challenging and stressful; however, succeeding with behavior change is empowering and increases confidence to take on new challenges.

**Fig 1:**
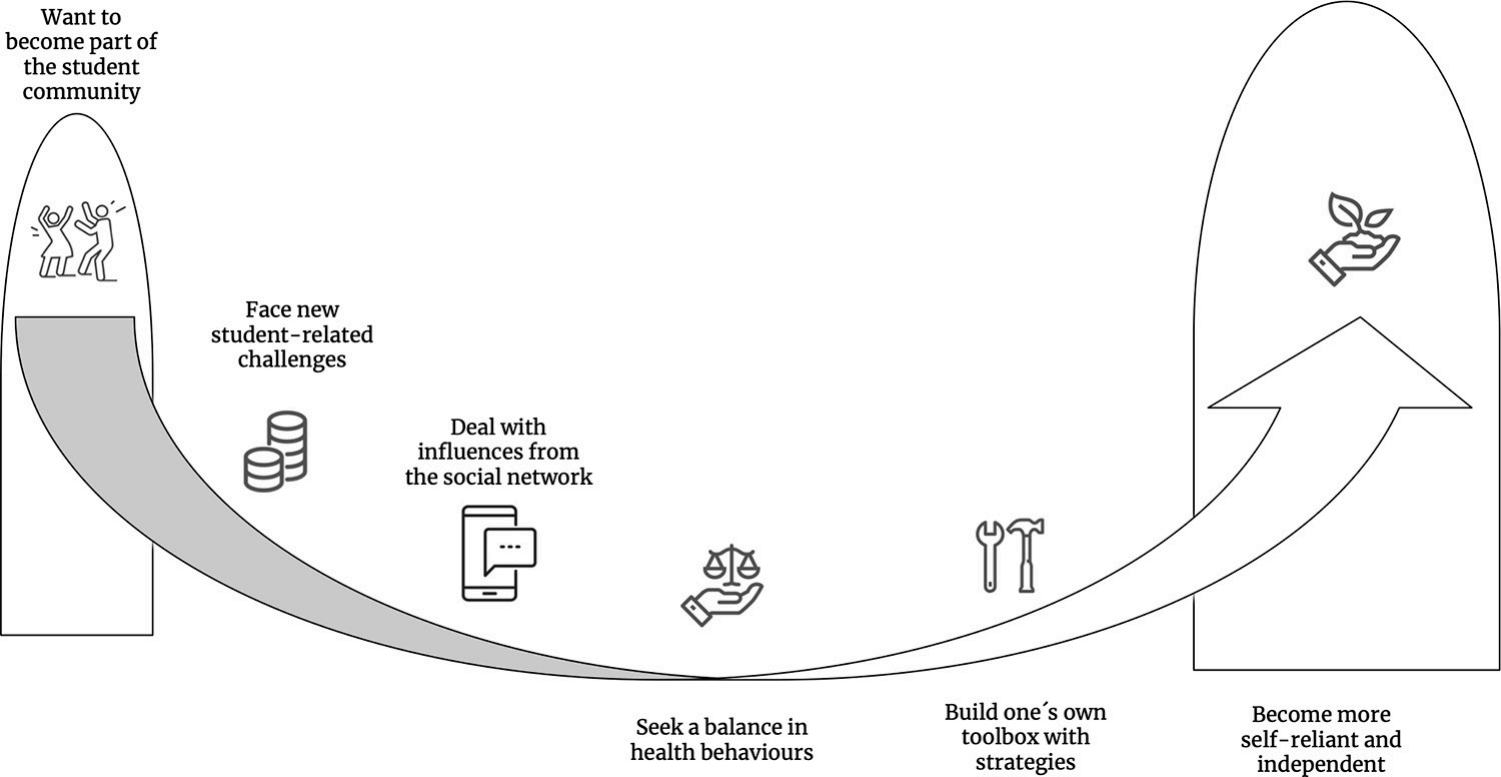
Illustration of the comprehensive understanding: Interview participants’ experiences of the balancing act.

As one progresses in academic life, health becomes more holistic, which influences the choice of strategies one uses to maintain and improve health. One becomes less influenced by peers and social networks, or by society in general, but more independent and self-reliant with respect to health-related behaviors and choices. An illustration of the comprehensive understanding is illustrated in Fig 1. The illustration can be read from left to right, indicating (to the far left), that junior students adapt and want to become part of the student community, for instance by consuming alcohol at parties. In addition, new student-related challenges such as a living on a limited budget are faced, but the students deal with influences from their social network, social media, and health norms of society. As time passes–moving to the right in the illustration–students transition and progress on a slack line through student life, seeking a balance in health behaviors. They build their toolbox with health behavior change strategies, and thus become more self-reliant and independent.

## Discussion

The findings signify that university students’ attitudes to health and health-related behaviors form a trajectory influenced by both internal and external elements. Hence, the discussion will primarily address aspects of i) finding one’s health, ii) finding one’s strategies, and iii) the transition of becoming a university student. Most importantly, the findings are developed in view of the walk on the slack line that the striving for health and healthy behaviors can represent for university students, and how this can be facilitated–or not–by contemporary and future attempts to develop and provide support.

### Finding one’s health

University students’ views on and experiences of health are strongly influenced by their current life situation. In addition, the academic environment and students’ health seem to be closely related. For instance, a study by Porru et al. [32] showed that students with greater exposure to student life challenges, including an unsupportive climate among peers and a high workload, report poorer mental and self-rated health. Students in this study were aware of the value of behaviors that have been found to be important for one’s health, including not drinking too much alcohol, eating healthily, being physically active, and not smoking. However, they reflected that their finances, study-stress, and sleeping behaviors strongly affected their everyday health and well-being. This indicates that behaviors commonly targeted by lifestyle behavior interventions [33] may not be on university students’ agenda.

In line with previous studies, our results suggest that university students prioritize academic success over health and well-being. For instance, Aceijas et al. [34] interviewed undergraduate students in the UK, showing that external factors such as economic hardship and academic pressure made healthy living a challenge in everyday life. In addition, Warnick et al. [35] showed that students at an elite college in the USA described a tendency not to prioritize health, and health was not articulated as important unless it impaired one’s ability to live one’s life. However, in contrast to our findings, the students in the study by Warnick et al. also considered that unhealthy lifestyle behaviors led to increased academic success.

### Finding one’s strategies

Students’ perspectives on health affect their choice of strategies to maintain or improve their health. Although lifestyle behaviors were not ‘front of mind’, and good health was taken for granted by the students, health was still valued as a fundamental prerequisite in life. The students in this study had experience of developing individual strategies to improve their health, such as implementing change in manageable steps, seeking social support, and planning. These strategies are common in behavior-change interventions, and can be found in taxonomies of behavior change techniques [36]. But the students also applied a strategy of seeking to find a balance between healthy behaviors and behaviors that might have a negative impact on their health in the long run; for instance, drinking with friends to feel good in the present and to cope with stress and academic demands. This balancing of behaviors was also found by Aceijas [34], who reported that students described smoking and alcohol as stress-relieving strategies. On the other hand, healthy lifestyle behaviors were known by the students to be a strategy that would help them to perform and succeed in their studies; for example, to gain more energy and avoid negative consequences, such as concentration difficulties or poor sleep. Students were aware of a norm regarding a healthy lifestyle in both social media and society at large, and stated that this norm sometimes affected them negatively, triggering a sense of guilt and stress from knowing that they were not living according to the perceived health standard. Being exposed to health messages on social media, for example, can both work as a source of inspiration for behavior change, but can also trigger health-related stress among students, regardless of whether they follow the advice or not, and providing students with health-related information could therefore raise ethical concerns [37].

### The transition of becoming a university student

Being a student and finding strategies to maintain one’s health can be perceived as a balancing act, a transition achieved by means of walking on a slack line. Meleis [38] describes transitions as life-changing experiences characterized by flow and movement over time, which can be defined as a passage or movement from one state or place to another. Transitions often require the individual to redefine themselves in relation to their new social context and come with the development of new knowledge and changed behavior. Another example of student transitions is provided by Wrench et al. [39], who asked first-year students at an Australian university how the transition to university life had affected their well-being and understanding of health. In agreement with this study, the students described how the transition had had a negative impact on their health behaviors, such as a poorer diet, decreased amounts of sleep, and increased stress levels, and students described how they both turned to friends for social support and also used personal strategies, such as planning and positive thinking, to deal with stress.

Further research that can provide insights into useful behavior change strategies for university students is needed, because in the past, interventions have been designed to only address university students’ behavior strategies to a certain extent. Future attempts to develop and deliver support for lifestyle behavior change could benefit from focusing on both the nature of lifestyle behaviors while transitioning through university and developing an improved understanding of the life-changing experiences that come with these transitions. With tailored health interventions to guide and empower them to improve their health, students can become better prepared to face the demands of student life.

### Strengths and limitations

The dependability of our study was strengthened by following the methodological steps described by Lindseth and Norberg [27, 29] and Elo and Kyngäs [31]. A strength of the study was that participants were recruited irrespective of whether they were seeking to alter their lifestyle behaviors, or not. Yet, acknowledging that self-selected participants might have introduced selection bias, this study, and its research question, necessitated interviewees that were able to elaborate on health behaviors, that is, had lived experience to share. The risk of self-selection was deemed acceptable since the study neither evaluated the prevalence of unhealthy behaviors, nor the type of health behaviors. The majority of participants were female students from the medical faculty, who were potentially more interested in the research topic, which could affect the transferability of the results. Studies including universities from other geographical areas outside Sweden would be of interest for future research. It is not certain that the richness of data is increased with a greater number of participants or more pages of text [40], but our experience was that the participants spoke with engagement, leading to no fewer than 405 pages of transcribed text.

Due to the Covid-19 pandemic, all interviews were conducted via internet link instead of in person. Even though Sweden did not have as harsh lock-downs as some countries, social isolation, distance education and digital meetings were still advocated across all universities. However, all students in this study were recruited at a time when the university was fully open and accessible. The interviews were conducted during this time period, and the students largely referred to their regular life, neither voluntarily addressing nor prompted to consider the pandemic.

Based on student preferences, both individual interviews and a group interview were conducted, and the data was analyzed together. These two approaches were similar, using the same open-ended questions within the one interview guide. However, focus groups may rely on group dynamics to stimulate discussion, while individual interviews are more likely to produce rich detail in understanding unique experiences [41]. To strengthen the study’s credibility, authors with various different research and professional competences in public health and qualitative methods were involved in the analysis. The first author performed the analysis, in close reflection and discussion with the co-authors (ACE, MB) in order to reach agreement on study categories and abstraction level [42], in accordance with the hermeneutic circle [27].

## Conclusions

Experiences of health and health-related behaviors while becoming and being a university student can be described as a transition–a balancing act of walking on a slack line–during which students seek a healthy balance to manage student life.

This study improves our understanding of students’ needs within the topic of health and lifestyle behavior change during the academic years. During their transition, students’ perceptions of health are modified, and personal strategies are gradually developed and refined as students become more experienced and self-reliant. The study offers a further understanding, imparting that healthy behavior and behavior change among university students, is fashioned in the flux of the constantly ongoing student life–where one needs to address and relate to behavior change as ‘a never ending process’.

Future public health interventions aimed at supporting students’ health and well-being should focus on the transitions and challenges of student life in order to be able to guide university students to improve their health.

## Supporting information

S1 File Interview guide. (DOCX)
