# Supplementary material for: A balancing act–finding one´s way to health and well-being: A qualitative analysis of interviews with Swedish university students on lifestyle and behavior change
Source: PLoS One. 2022 Oct 13;17(10):e0275848. doi: 10.1371/journal.pone.0275848 (PMC9560508; doi:10.1371/journal.pone.0275848)
Supplement: S1 File — (DOCX) [file pone.0275848.s001.docx]

# Interview guide

There are a few details that I’d like to ask to start with… what educational program are you following? (…) what is your year of birth? (…) how do you define your gender? (…)

This research is about *lifestyle* and *lifestyle behaviors.* These are two words that we hear quite often and might sometimes even use ourselves without thinking much about what they really mean…. If I start by saying the word ‘lifestyle’ – tell me, what do you think of?

The other phrase which is key to our dialogue is ‘lifestyle behaviors’. What comes to mind when you hear the phrase ‘lifestyle behaviors’?

So far, we have talked about ‘lifestyle’ and ‘lifestyle behaviors’ as concepts…but lifestyle and lifestyle behaviors are also something that we can change – if we want to (…) which we may all have experiences of – and it can be either easy or hard, and to different extents, to make such changes… This study is about changing lifestyle behaviors – please tell me your thoughts about that [changing lifestyle behaviors]?

OK – so now we’ve talked about lifestyle behaviors and behavior change in general, but I’d like for you to consider yourself a bit more; what about you and your life… Have you ever made any adjustments or changes to your lifestyle behaviors? (…) Which behavior(s), and how did it work out?

Thanks, these were the issues I wanted you to consider… When participating in such an interview, sometimes thoughts pop up after a while. (…) Is there anything you’ve begun to think about that you would like to share or ask?

… means short pause

(…) means longer pause

– means short pause and emphasizing the next word

[ ] means word is implied, and perhaps not spoken
